# Targeting p53-deficient chronic lymphocytic leukemia cells *in vitro* and *in vivo* by ROS-mediated mechanism

**DOI:** 10.18632/oncotarget.12110

**Published:** 2016-09-19

**Authors:** Jinyun Liu, Gang Chen, Helene Pelicano, Jianwei Liao, Jie Huang, Li Feng, Michael J. Keating, Peng Huang

**Affiliations:** ^1^ Department of Translational Molecular Pathology, The University of Texas MD Anderson Cancer Center, Houston, Texas 77030, USA; ^2^ Sun Yat-Sen University Cancer Center, State Key Laboratory of Oncology in South China, Collaborative Innovation Center for Cancer Medicine, Guangzhou 510060, China; ^3^ Department of Leukemia, The University of Texas MD Anderson Cancer Center, Houston, Texas 77030, USA

**Keywords:** CLL, oxidative stress, p53, PEITC

## Abstract

Chronic lymphocytic leukemia (CLL) is the most common adult leukemia in Western countries. Loss of p53 function in CLL cells due to chromosome 17p deletion or p53 mutations often leads to a more malignant disease phenotype and is associated with drug resistance and poor clinical outcome. Thus, development of novel therapeutic strategies to effectively target CLL cells with p53 deficiency is clinically important. Here we showed that p53-null CLL cells were highly sensitive to ROS-mediated cell killing due to their intrinsic ROS stress. We further demonstrated that a natural compound phenethyl isothiocyanate (PEITC) was able to effectively kill CLL cells with loss of p53, even under the protection of stromal cells. In p53-defficient CLL cells, PEITC induced a rapid depletion of glutathione and a severe accumulation of ROS, leading to massive leukemia cell death in the stromal microenvironment. The drug-induced cell death was associated with a significant decrease of in MCL-1 survival molecule. We further showed that ROS-mediated cell death was the key mechanism by which PEITC induced cytotoxicity, since such cell death could be prevented by addition of antioxidant NAC. Importantly, *in vivo* study showed that PEITC was able to induce substantial leukemia cell death in mice. Treatment of CLL mice harboring *TCL1-*Tg:*p53^−/−^* genotype with PEITC significantly prolonged the median survival time of the animals. Our study identifies a vulnerability of p53-null CLL cells with high sensitivity to ROS-generating agents, and suggests that PEITC may potentially be useful for clinical treatment of CLL with 17p deletion and p53 mutations.

## INTRODUCTION

Chronic lymphocytic leukemia is characterized by the accumulation of dysfunctional B lymphocytes in the blood, bone marrow, spleen and other organs [1]. In the recent years, major progress has been made in our understanding of CLL biology and in the development of new therapeutic agents, which significantly improve the clinical outcomes of CLL patients. However, CLL still remains as an incurable disease [2]. Cytogenetic alterations are commonly observed in CLL patients, and certain cytogenetic changes are associated with aggressive disease progression and poor prognosis [3]. Notably, deletion of chromosome 17p renders the leukemia cells more resistant to standard therapy, and is associated with more aggressive disease progression and a significantly shorter overall survival [4]. Chromosome 17p contains the p53 gene that encodes the tumor suppressor p53 protein. It has been suggested that a loss of p53 function in CLL cells with 17p-deletion maybe a major factor contributing to the drug resistance and poor clinical outcome of this subgroup of patients [5–6]. Loss of p53 function has been documented in many cancer types [7]. Although 17p-deletion is seen in approximately 5–10% of CLL patients, mutations in p53 genes are observed in approximately 30% of CLL patients [8, 9]. This suggests that a large proportion of CLL patients likely have a defect in p53 function. As the guardian of genome, p53 plays a pivotal role in regulating important cellular functions such as DNA damage response, cell cycle regulation, apoptosis, and drug sensitivity. However, the exact mechanisms by which a loss of p53 contributes to drug resistance and disease progression in CLL remains to be elucidated. Recent study suggested that one of the mechanisms was through up-regulation of MCL-1 expression *via* suppression of microRNA-15a/miR-16-1 [10].

Considering the important role of loss of p53 in cancer development and drug resistance in CLL cells, it is important to develop new therapeutic strategies that are effective in eliminating p53-null CLL cells based on their biological properties. One noticeable biochemical feature of CLL cells is their intrinsic high ROS stress [11–13], which renders them more dependent on cellular antioxidants such as GSH to maintain the redox balance. As such, the high oxidative stress could serve as a biochemical basis to preferentially target CLL cells, using proper redox-modulating strategies [14]. For instance, recent studies showed that phenethyl isothiocyanate (PEITC), a natural compound found in certain vegetables, could induce depletion of glutathione (GSH) and cause severe ROS accumulation leading to massive death of CLL cells [13, 15]. PEITC seems able to effectively kill fludarabine-resistant CLL cells [13]. Importantly, p53 plays a significant role in maintaining mitochondrial integrity and metabolic functions [16, 17] and also exhibits an antioxidant function [18]. Thus, a loss of p53 function due to mutations or 17p-deletion in CLL cells would be expected to cause mitochondrial dysfunction and subsequently disrupt redox homeostasis, leading to increased ROS generation and oxidative stress.

Based on the above observations, we hypothesized that CLL cells with loss of p53 function might be more vulnerable to further oxidative stress, and targeting ROS stress might be an attractive therapeutic strategy for treatment of CLL with 17p-deletion and/or p53 mutations. The main goal of this study was to test the possibility to use PEITC as a potential agent to effectively eliminate CLL cells with loss of p53, using both *in vitro* assay with primary leukemia cells isolated from CLL patients with 17p-deletion and *in vivo* test in a CLL mouse model with *TCL1*-Tg:*p53*^−/−^ genotype [10]. We demonstrated that PEITC was highly effective in eliminating p53-defective CLL cells, even in the presence of bone marrow stromal cells, which usually protect CLL cells from the cytotoxic effect of conventional chemotherapeutic agents. *In vivo*, PEITC exhibited significant therapeutic effects and significantly improved the overall survival of the p53^−/−^ CLL mice.

## RESULTS

### Effective killing of primary CLL cells with 17p-deletion by PEITC in the presence of bone marrow stromal cells

**Figure 1:**
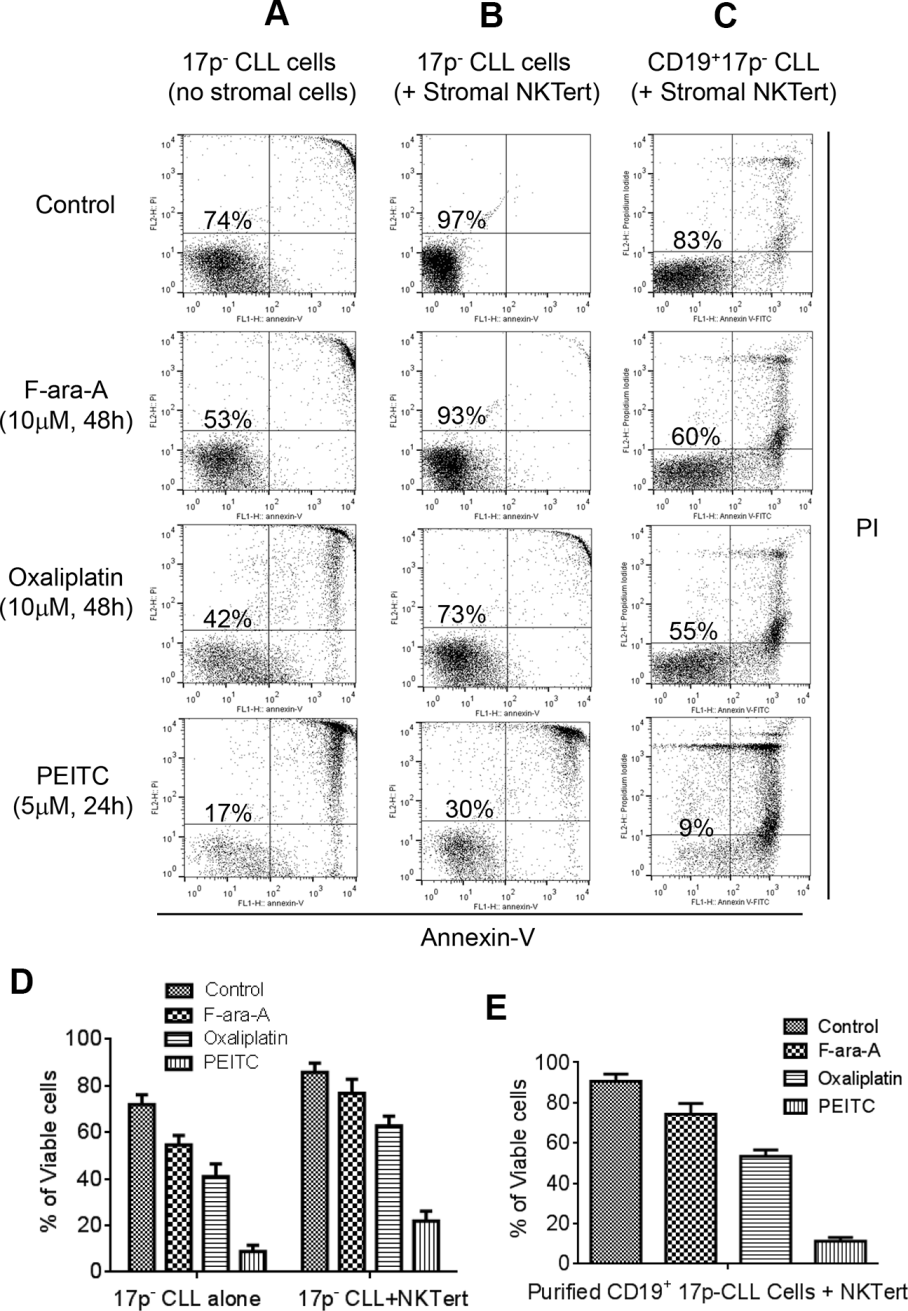
Comparison of cytotoxic effect of PEITC and standard chemotherapeutic agents in primary CLL cells with 17p deletion (**A**) Cell death induced by F-ara-A (10 μM, 48 h), Oxaliplatin (10 μM, 48 h), or PEITC (5 μM, 24 h) in primary 17p- CLL cells cultured alone (without stromal cells). Cell viability was analyzed by flow cytometry after double staining with Annexin V-PI. Representative dot plots of independent experiments using 9 different CLL patient samples are showed (*n* = 9). (**B**) Cell death induced by F-ara-A (10 μM, 48 h), Oxaliplatin (10 μM, 48 h), or PEITC (5 μM, 24 h) in 17p- CLL cells co-cultured with human bone marrow stromal NKTert cells. Cell viability was analyzed by flow cytometry after double staining with Annexin V-PI. Representative dot plots of independent experiments using 9 different CLL patient samples are showed (*n* = 9). (**C**) Cell death induced by F-ara-A (10 μM, 48 h), Oxaliplatin (10 μM, 48 h), or PEITC (5 μM, 24 h) in purified 17p- CD19+ CLL cells co-cultured with human bone marrow stromal NKTert cells. Cell viability was analyzed by flow cytometry after double staining with Annexin-V/PI. Representative dot plots of 3 independent experiments using 3 different CLL patient samples are showed (*n* = 3). (**D**) Quantitative comparison of cell death induced by F-ara-A (10 μM, 48 h), Oxaliplatin (10 μM, 48 h), or PEITC (5 μM, 24 h) in 17p- CLL cells alone or co-cultured with NKTert cells. (**E**) Quantitative comparison of cell death induced by F-ara-A (10 μM, 48 h), Oxaliplatin (10 μM, 48 h), or PEITC (5 μM, 24 h) in purified 17p- CD19+ CLL cells co-cultured with NKTert cells.

We first tested the cytotoxic effect of PEITC against primary CLL cells with 17p deletion isolated from the peripheral blood samples of CLL patients. Two standard anti-CLL drugs, Fludarabine (F-ara-A, the active chemical form of Fludarabine for *in vitro* study) and Oxaliplatin, were used for comparison with PEITC. As shown in Figure 1A, primary CLL cells with 17p-deletion were relatively resistant to F-ara-A and Oxaliplatin at a high drug concentration (10 μM). There were 53% and 42% survival cells at 48 h after treatment with F-ara-A and Oxaliplatin, respectively. In contrast, PEITC at a relatively low concentration (5 μM) effectively killed 17p- CLL cells, with only 17% viable cells remained at 24 h after drug incubation. The resistance of 17p- CLL cells to standard anti-CLL agents and high sensitivity to PEITC were consistently observed in separate experiments with 9 different CLL patient samples (Figure 1D).

Since stromal tissue microenvironment plays a major role in protecting cancer cells, we then compared the ability of the three compounds to kill 17p- CLL cells in co-culture with human bone barrow stromal cells (NKTert). As shown in Figure 1B, 17p- CLL cells co-cultured with stromal cells exhibited better survival (97% viable cells without drug treatment), and became more resistant to F-ara-A (10 μM) and Oxaliplatin (10 μM) with 93% and 73% viable cells at 48 h, respectively. Similarly, 17p- CLL cells co-cultured with stromal cells were also resistant to another chemotherapeutic agent Bendamustine (Supplementary Figure S1). Under this stromal co-culture condition, PEITC (5 μM) was still effective against 17p- CLL cells, leading to a loss of 70% cell viability in 24 h. Interestingly, PEITC showed similar efficacy against CLL cell without 17p-deletion (Supplementary Figure S2), suggesting that this compound is equally effective in CLL cells regardless of the p53 status.

We also used purified CD19+ CLL cells form patient samples to further confirm the above observations. CD19+ B-cells were isolated from three 17p- CLL blood samples, using CD19 antibody-coated microbeads. The purified CLL cells were then incubated with F-ara-A, Oxaliplatin, or PEITC in co-culture with stromal cells. As shown in Figure 1C and 1E, the purified CD19^+^ CLL cells with 17p deletion were relatively resistant to F-ara-A and Oxaliplatin (60% and 55% survival, respectively), but remained highly sensitive to PEITC (9% survival).

We also tested if PEITC could induce autophagy, which could play a role protecting cells under certain stress conditions. CLL cells isolated from four different CLL patient samples were incubated with various concentrations (1–20 μM) of PEITC for 24 h, and Western blotting was performed to analyze the expression of autophagy marker LC3. As shown in Supplementary Figure S3, PEITC did not induced any significant changes in LC3, suggesting that this compound did not induce significant autophagy in CLL cells.

### Cytotoxic effect of PEITC against p53^−/−^ leukemia cells from mice *in vitro* and *in vivo*

**Figure 2:**
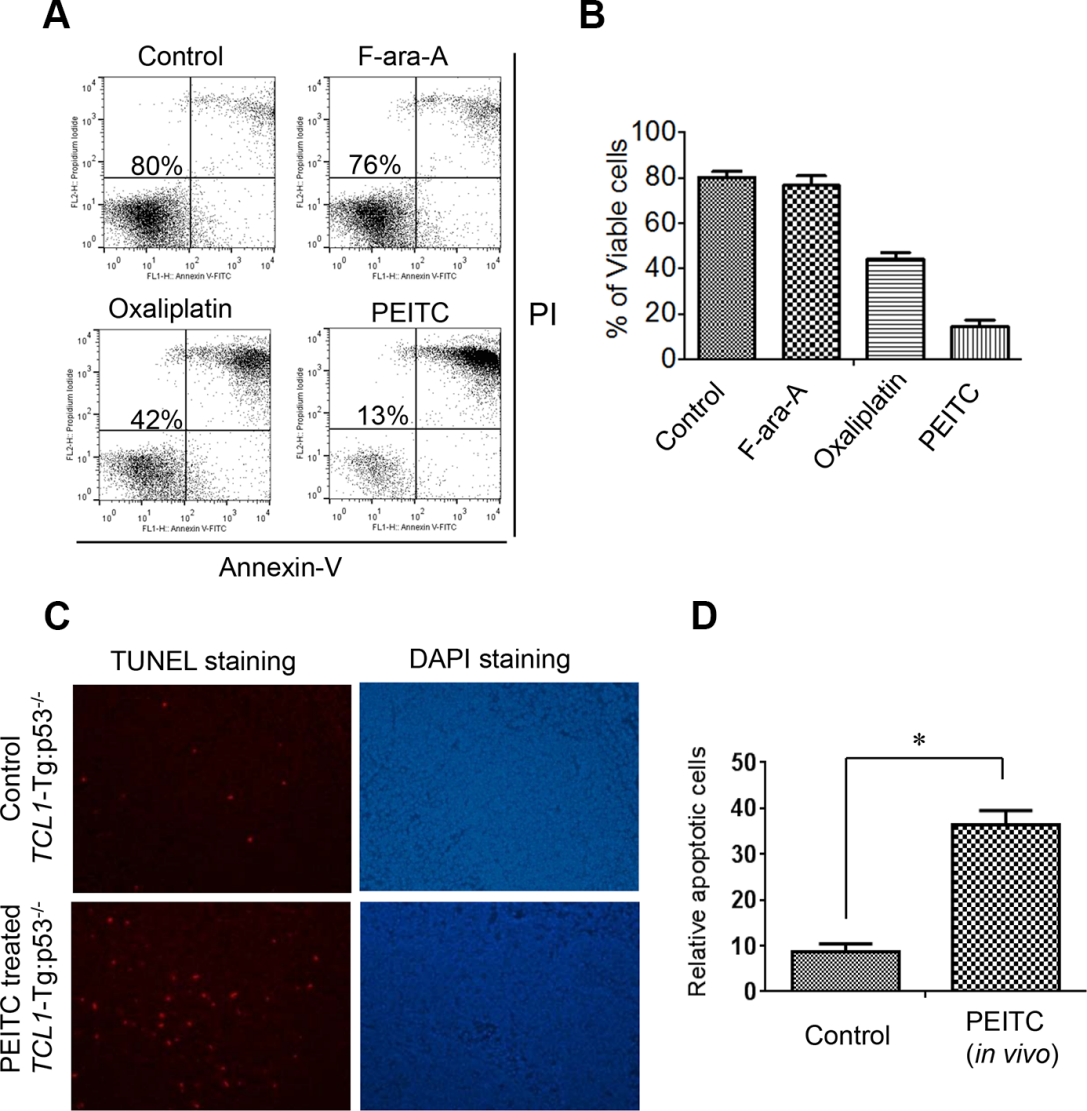
Effective killing of mouse leukemic cells by PEITC *in vitro* and *in vivo* (**A**) Cell death induced by F-ara-A (10 μM, 48 h), Oxaliplatin (10 μM, 48 h), or PEITC (5 μM, 24 h) in leukemic splenocytes isolated from CLL mice with *TCL1*-Tg:p53^−/−^ genotype. The isolated leukemia cells from a mouse spleen were co-cultured with mouse stromal Kusa-H1 cells and incubated with the drugs as indicated, and cell viability was analyzed by flow cytometry after double staining with Annexin V-PI. Representative dot plots of 6 independent experiments using 6 *TCL1*-Tg:p53^−/−^ mice are showed (*n* = 6). (**B**) Quantitative comparison of cell death induced by F-ara-A (10 μM, 48 h), Oxaliplatin (10 μM, 48 h), or PEITC (5 μM, 24 h) in isolated mouse leukemic cells in the presence of mouse stromal Kusa-H1 cells. (**C**) Apoptotic cells detected by TUNEL assay in the spleen tissues isolated from PEITC treated *TCL1*-Tg:p53^−/−^ mice (*n* = 3). (**D**) Quantitative comparison of apoptotic cells in the spleen isolated from the control and PEITC treated mice with *TCL1*-Tg:p53^−/−^ genotype, *p < 0.05.

We next used a CLL mouse model with *TCL1*-Tg:*p53*^−/−^ genotype to further test the ability of PEITC to kill leukemia cells with defined p53 deletion *in vitro* and *in vivo*. We previously showed that this mouse model resembles human CLL patients with 17p-deletion with aggressive disease progression and drug-resistant phenotype [10]. Leukemic lymphocytes were isolated from the spleens of *TCL1*-Tg:*p53*^−/−^ mice (age 4-month) and co-cultured with mouse stromal cells (Kusa-H1 cells). The cells were treated with 5 μM PEITC for 24 h, 10 μM of F-ara-A for 48 h, or 10 μM Oxaliplatin for 48 h, and then analyzed by cell viability assay. We found that these cells were resistant to F-ara-A treatment (76% viable cells compared to 80% viable cells in the untreated control sample) and partially resistant to Oxaliplatin (42% viable cells). They were also resistant to Bendamustine (Supplementary Figure S1). In contrast, the mouse leukemia cells exhibited massive cell death when incubated with a relatively low concentration (5 μM) of PEITC, resulting in a loss of 87% cells in 24 h (Figure 2A, 2B). Thus, consistent with the observations in human CLL cells with 17p deletion, the leukemic cells from the spleens of *TCL1*-Tg:*p53*^−/−^ mice were also resistant to traditional chemotherapeutic agents but were highly sensitive to PEITC *in vitro*.

To test the ability of PEITC to induce leukemia cell death *in vivo*, we treated *TCL1*-Tg:p53^−/−^ mice with PEITC by intravenous injection. After treatment for 2 weeks, the mouse spleen tissue sections were processed for analysis of cell death using a terminal deoxynucleotidyl transferase dUTP nick end labeling (TUNEL) assay. As shown in Figure 2C–2D, there was a significant increase in TUNEL signal in the spleens of mice treated with PEITC, indicating that PEITC was able to induce apoptotic cell death in leukemic splenocytes *in vivo*.

### Induction of severe ROS accumulation and glutathione depletion by PEITC in p53-deficient CLL cells

**Figure 3:**
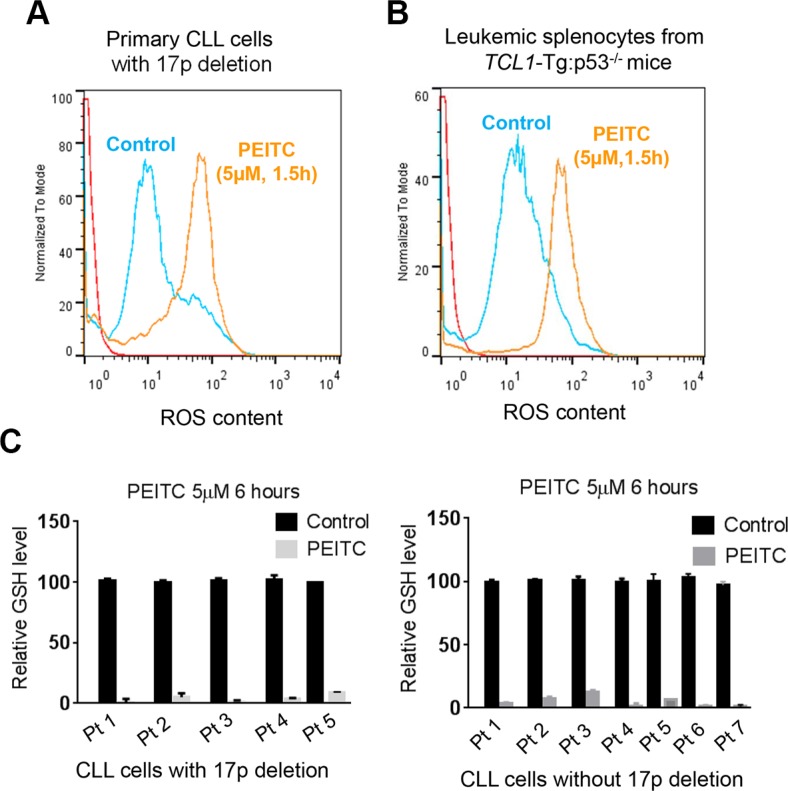
ROS accumulation and glutathione depletion induced by PEITC in p53-deficient CLL cells (**A**) ROS generation induced in 17p- CLL cells incubated with 5 μM PEITC for 1.5 h (*n* = 6). Cellular ROS was analyzed by flow cytometry after staining with DCF-DA. (**B**) ROS induced in mouse splenocytes from CLL mice with *TCL1*-Tg:p53^−/−^ genotype. Cells were treated with 5 μM PEITC for 1.5 h (*n* = 4), and cellular ROS was analyzed by flow cytometry after staining with DCF-DA. (**C**) Relative GSH levels measured in primary CLL cells with 17pdeletion (*n* = 5, left panel) or CLL cells without 17p-deletion (*n* = 7, right panel) after treatment with 5 μM PEITC for 6 hours. Solid black bars, control cells without PEITC treatment; gray bar, PEITC-treated cells.

Based on the previous observations that PEITC could induce ROS accumulation by causing GSH depletion in cancer cells [13, 21], we tested if this mechanism might be responsible for killing CLL cells with loss of p53. We isolated primary leukemia cells from CLL patients with 17p-deletion and leukemic splenocytes from *TCL1*-Tg:*p53*^−/−^ mice, and incubated them with PEITC. Shortly after drug incubation, intracellular ROS was measured by flow cytometry, which revealed a substantial increase of cellular ROS in the PEITC-treated cells 90 minutes after PEITC treatment. This was consistently observed in both human primary CLL cells (Figure 3A) and in mouse leukemic splenocytes (Figure 3B). Measurement of GSH showed a severe GSH depletion 6 hours after PEITC treatment in all five CLL patient samples with 17p-deletion and seven CLL samples without 17p-deletion (Figure 3C). It is important to note that these biochemical changes occurred within several hours of drug incubation and were prior to cell death. Thus, induction of GSH depletion and severe ROS accumulation are upstream events that likely result in severe oxidative damage leading to massive death of the leukemia cells.

**Figure 4:**
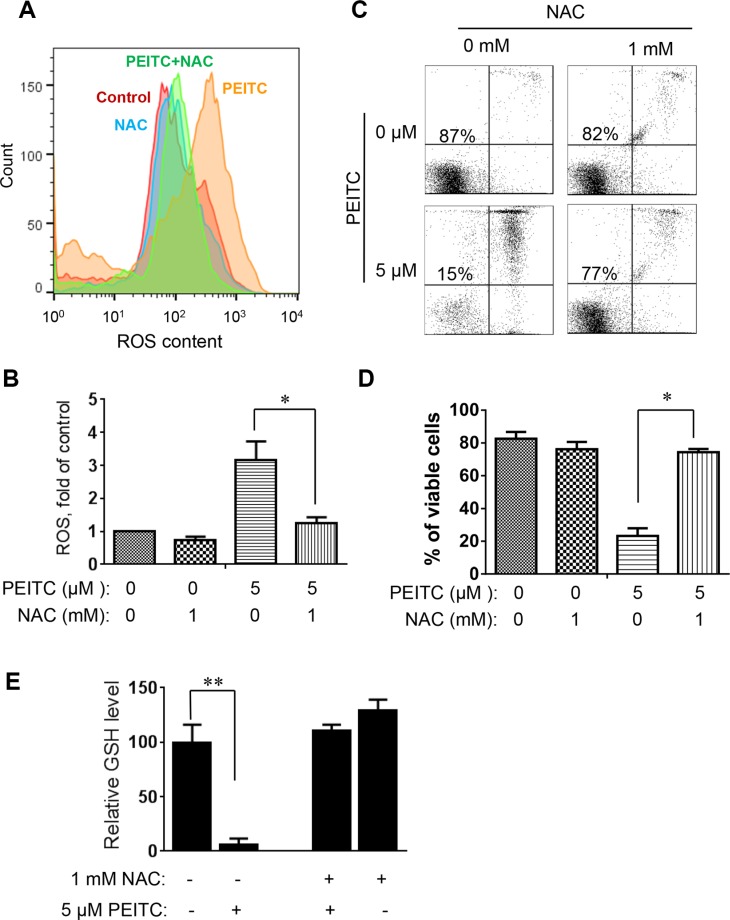
Effect of NAC on PEITC-induced ROS accumulation and cell death in primary CLL cells with 17p-deletion (**A**) Effect of NAC on PEITC-induced ROS accumulation in primary 17p- CLL cells. 17p- CLL cells were treated with 1 mM NAC for 1 hour before exposure to 5 μM PEITC for 2 h. Cellular ROS was analyzed by flow cytometry after staining with DCF-DA. Data shown are representative of 6 independent experiments. (**B**) Bar graphs showing quantitative analysis of ROS in CLL cells treated with PEITC, NAC, and their combination. *p < 0.05 (*n* = 6). (**C**) Effect of NAC on PEITC-induced cell death in primary CLL cells 17p- CLL cells with 17p-deletion were treated with 1 mM NAC for 1 hour before exposure to 5 μM PEITC for 24 h. Cell viability was analyzed by flow cytometry after double staining with Annexin V-PI. (**D**) Bar graphs showing quantitative analysis of cell death in primary CLL cells treated with PEITC, NAC, and their combination. *p < 0.05 (*n* = 6). (**E**) Depletion of cellular glutathione by PEITC treatment (5 μM, 6 h) in primary CLL cells with 17p-deletion in the presence or absence of 1 mM NAC. **p < 0.01 (*n* = 6).

**Figure 5:**
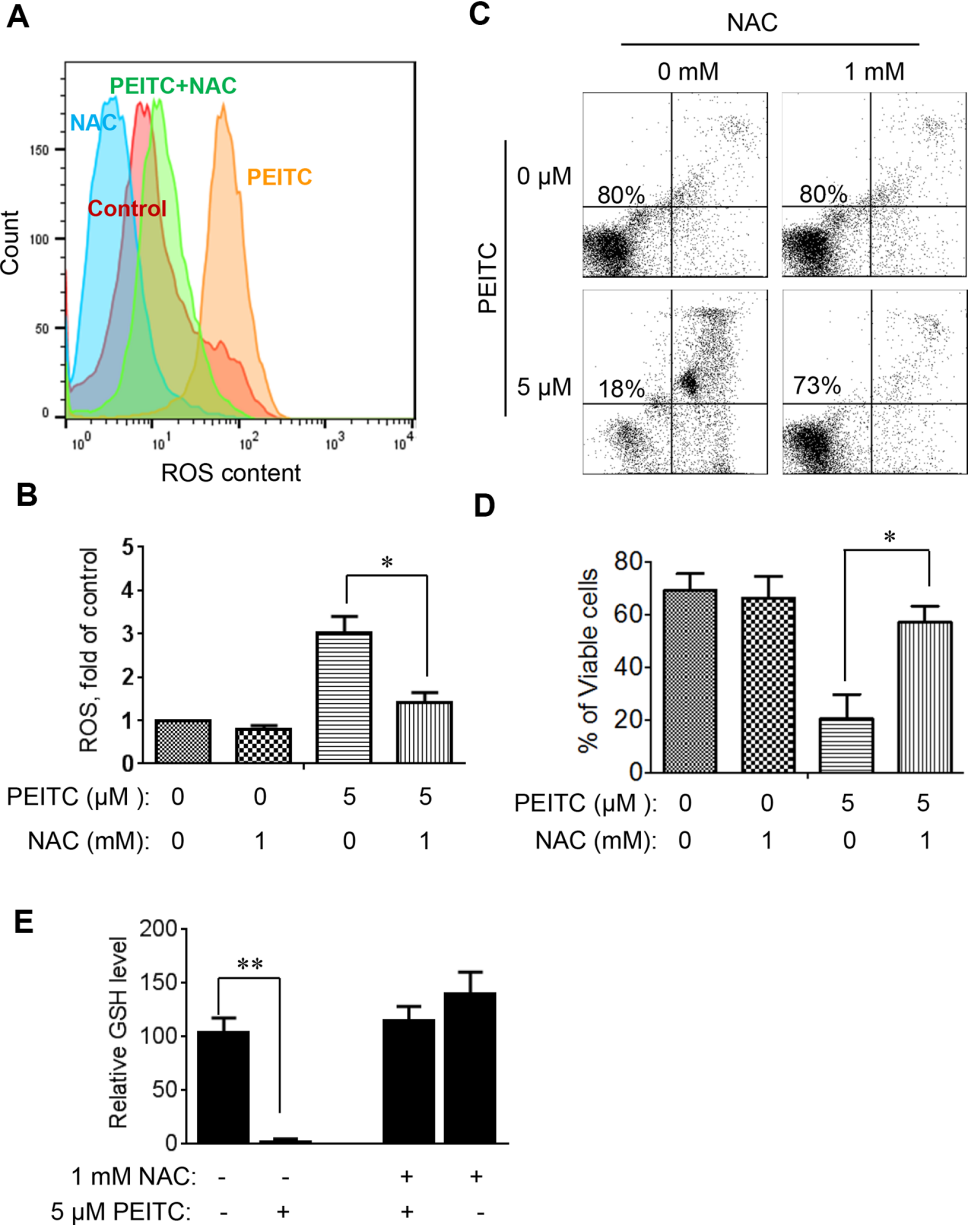
Effect of NAC on PEITC-induced ROS accumulation and cell death in mouse leukemia cells isolated from the spleen of mice with *TCL1*-Tg:p53^−/−^ genotype (**A**) Effect of NAC on PEITC-induced ROS accumulation in p53-null mouse leukemic cells. The p53^−/−^ mouse leukemic cells were treated with 1 mM NAC for 1 hour before exposure to 5 μM PEITC for 2 hours. Cellular ROS was then analyzed by flow cytometry after staining with DCF-DA. (**B**) Bar graphs showing quantitative analysis of ROS in mouse leukemia cells treated with PEITC and/or NAC as indicated. *p < 0.05 (*n* = 6). (**C**) Effect of NAC on PEITC-induced cell death in in p53-null mouse leukemic cells. Cells were treated with 1 mM NAC for 1 h before exposure to 5 μM PEITC for 24 h. Cell viability was analyzed by flow cytometry after double staining with Annexin V-PI. (**D**) Bar graphs showing quantitative analysis of cell death in mouse leukemia cells. *p < 0.05 (*n* = 6). (**E**) Depletion of cellular glutathione by PEITC treatment (5 μM, 6 h) in *TCL1*-Tg:p53^−/−^ splenocytes in the presence or absence of 1 mM NAC. **p < 0.01 (*n* = 6).

To further evaluate whether induction of GSH depletion and severe ROS stress were primary events that triggered cell death in CLL cells with loss of p53, primary CLL cells with 17p- deletion were treated with an antioxidant NAC (which promotes glutathione synthesis), PEITC, or their combination. NAC consistently prevented PEITC-induced ROS accumulation (Figure 4A–4B), GSH depletion (Figure 4E) and cell death (Figure 4C–4D) in different CLL patient samples with 17p-deletion. Similar results were observed in mouse leukemic splenocytes isolated from *TCL1*-Tg:p53^−/−^ mice (Figure 5). These data together suggest that PEITC induced cell death in both human and mouse CLL cells with loss of p53 through depletion of GSH and induction of severe ROS stress.

### Rapid degradation of MCL-1 protein induced by PEITC in 17p- CLL cells

**Figure 6:**
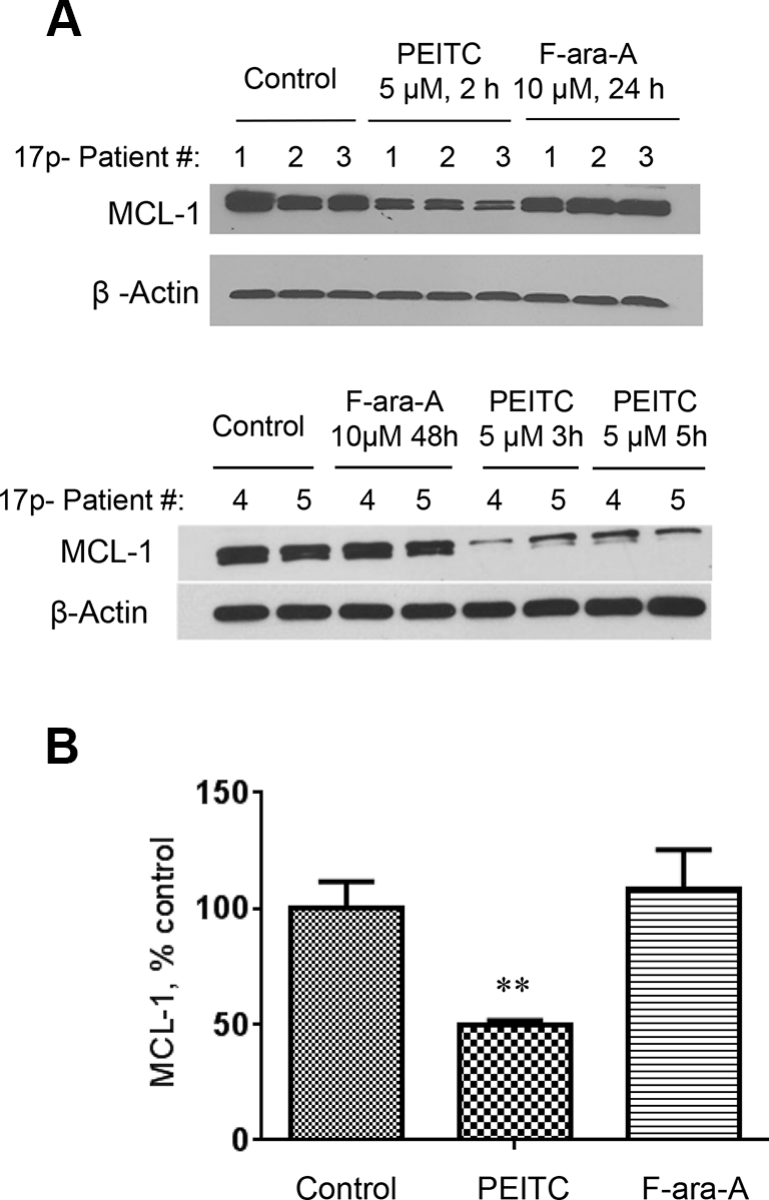
Decrease of MCL-1 protein induced by PEITC in CLL cells with 17p-deletion (**A**) MCL-1 protein levels were measured by Western Blotting in 17p- CLL cells before and after treatment with 5 μM PEITC for 2–5 h, or with 10 μM F-ara-A for 24–48 h. (**B**) Quantitative analysis of MCL-1 protein levels by Image J, **p < 0.01, between groups.

Because MCL-1 is a ROS-sensitive anti-apoptotic protein that plays an important role in the survival of CLL cells [13] and is up-regulated in primary 17p- CLL cells and leukemic splenocytes from *TCL1*-Tg:*p53*^−/−^ mice [10], we tested the possibility that PEITC might induce degradation of MCL-1 in 17p- CLL cells owing to its ability to induce severe ROS stress. As shown in Figure 6, when CLL cells with 17p-deletion were treated with 5 μM PEITC, there was a significant decrease in MCL-1 protein within 2 h and further decrease was observed as the incubation time prolonged. This is consistent with our previous finding that PEITC induced degradation of MCl-1 in part by causing deglutationylation of MCL-1 protein [13]. In contrast, incubation of 17p- CLL cells with 10 μM F-ara-A for 24–48 h did not cause any change in MCL-1 protein (Figure 6A).

### *In vivo* therapeutic activity of PEITC in CLL mice with p53 deletion

**Figure 7:**
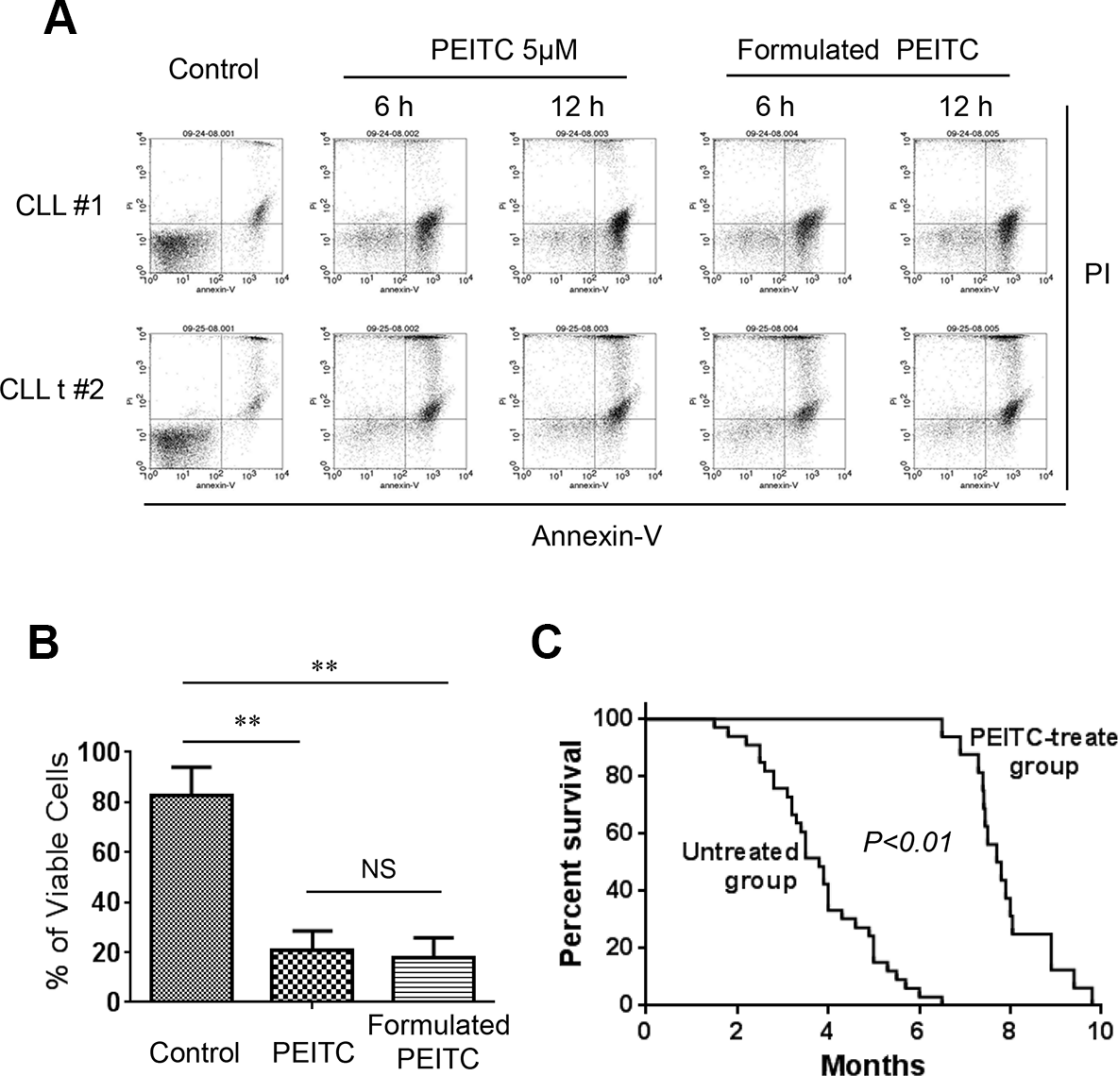
Formulated PEITC in pluronic F127 nanoparticles exhibited potent anti-CLL activity *in vitro* and *in vivo* (**A**) Formulated PEITC in pluronic F127 nanoparticles and original free PEITC exhibited similar cytotoxicity against CLL cells. Cell viability was analyzed by flow cytometry after double staining with Annexin V-PI. (**B**) Bar graphs showing quantitative analysis of cell death in mouse leukemia cells treated with 5 μM formulated PEITC and PEITC for 24 h. **p < 0.01. (**C**) Survival curve (Kaplan-Meier) of control *TCL1*-Tg:p53^−/−^ mice (untreated, *n* = 47), and mice treated with formulated PEITC- (*n* = 16). Median survival time for formulated PEITC treated *TCL1*-Tg:p53^−/−^ mice was 7.8 months compared to 3.5 months for untreated mice, *p* < 0.01 between the two groups.

Based on the observations that PEITC was able to induce significant CLL cell death in primary human CLL cells *in vitro* and in leukemic splenocytes in CLL mice, we further evaluated the *in vivo* therapeutic activity of PEITC in CLL mice with *TCL1*-Tg:p53^−/−^ genotype. PEITC was first formulated in a stable nanoparticle formulation using the pluronic F127 as the carrier as described in Methods. To ensure that such formulation did not cause loss of anticancer activity, we first compared the *in vitro* activity of the original PEITC and the formulated PEITC for their ability to kill both primary human CLL and mouse leukemic cells. As shown in Figure 7A–7B, the formulated PEITC and the original free PEITC exhibited comparable cytotoxicity against CLL cells *in vitro*.

We then evaluated the *in vivo* antitumor activity of the formulated PEITC in CLL mice with *TCL1*-Tg:*p53*^−/−^ genotype. These mice developed leukemia with aggressive disease progression, resembling human CLL disease with 17p deletion [10]. Without any drug treatment, over 50% of mice died from CLL at 4-month of age, and most mice died within 6 months (Figure 7C). Treatment with PEITC significantly prolonged the survival of CLL mice, with almost all mice survived over 6 months (Figure 7C). The median survival time in the PEITC-treated group was 7.8 months, compared to 3.5 months in the control group. In a separate study, we found that the traditional drug fludarabine exhibited only moderate therapeutic activity in the *TCL1*-Tg:*p*53−/− mice with a median survival time of 5.3 months (data not shown). These results together strongly suggest that PEITC was highly effective in killing p53-deficient CLL cells *in vitro*, and exhibited promising therapeutic activity *in vivo* in the *TCL1*-Tg:*p53*^−/−^ mouse model.

## DISCUSSION

Metabolic alterations including altered nutrient metabolism and elevated ROS generation are among the hallmarks of cancer [19]. Genetic and epigenetic alterations of p53 gene have been frequently observed in various types of cancer [20]. Such genetic alterations, through their downstream signaling processes, often induce metabolic changes in cancer cells. The tumor suppressor molecule p53 plays an important role in maintaining mitochondrial genetic integrity and normal metabolic functions, and a loss of p53 function has been linked to mitochondrial DNA (mtDNA) mutations and elevated ROS production in cancer cells [16–18]. Thus, a loss of normal p53 function in CLL cells due to gene mutations or deletion (17p-deletion) would lead to elevated oxidative stress, and render the leukemia cells more vulnerable to further ROS stress induced by pharmacological agents. This study was conducted to test this research hypothesis, using primary CLL cells from patients with 17p deletion and CLL mice with *TCL1*-Tg:*p*53^−/−^ genotype as experimental models. PEITC was used to induce ROS stress through its ability to cause rapid glutathione depletion, which is known to effectively kill K-Ras transformed ovarian cancer cells and F-ara-A-resistant CLL cells through a ROS-mediated mechanism [13, 15, 21]. The current study showed that PEITC is indeed effective in killing p53-null CLL cells, which are otherwise resistant to standard anti-CLL drugs such as F-ara-A, Oxaliplatin and Bendamustine.

The promising therapeutic activity of PEITC was observed both *in vitro* and *in vivo*. We further confirmed that the key mechanism of PEITC action against p53-null CLL cells was mediated by induction of a rapid depletion of GSH and severe accumulation of ROS. This was evidenced by direct measurement of GSH (Figure 3C) and by the ability of NAC, a glutathione precursor and antioxidant, to prevent PEITC-induced ROS accumulation and suppress the drug-induced cell death both in primary 17p- leukemia cells from CLL patients (Figure 4) and in mouse leukemic cells from *TCL1*-Tg:p53^−/−^ mice (Figure 5). It is worth noting that PEITC is equally effective in killing CLL cells without loss of p53 (Supplementary Figure S2), since CLL cells are in general under high ROS stress and highly dependent on GSH to maintain redox balance. The ability of PEITC to effectively kill CLL cells with or without 17p deletion would be advantageous as a drug for clinical treatment of CLL patients with 17p deletion, since these patients harbor both 17p-deleted and 17p-wild type CLL cells.

In the tumor microenvironment, the interactions between cancer cells and stromal cells are complex biological processes that, through secretion of stromal factors and metabolic communication, often promote tumor cell survival, proliferation, and migration, leading to drug resistance and metastasis [19]. For example, elevated ROS levels in p53-deficient fibroblasts may induce E-cadherin expression in tumor cells and thereby promote cancer cell migration [22]. In case of CLL, the stromal factors such as SDF-1 (CXCL-12), BAFF, and APRIL play a major role in protection of the leukemia cells [23]. Furthermore, stromal cells can also provide certain critical metabolites such as cysteine to CLL cells to enhance their GSH synthesis and thus promote CLL survival and drug resistance [24]. The stromal protection of leukemia cells is considered as an important factor contributing to the persistence of residual disease after chemotherapy, and imposes a major challenge in clinical treatment of CLL. Our study showed that PEITC was able to effectively kill p53-null CLL cells in the presence of bone marrow stromal cells, suggesting that this compound could be effective in the *in vivo* microenvironment. Indeed, our animal study showed that this compound was able to induce substantial apoptosis of leukemia cells *in vivo* (Figure 2C), and significantly prolonged the overall survival of CLL mice (Figure 7C), suggesting its potential utility for clinical treatment of CLL.

MCL-1 is an important anti-apoptotic molecule that belongs to the Bcl-2 family. Our study showed that PEITC was able to cause a rapid decrease in MCL-1 protein in p53-null CLL cells within 2–5 hours after drug incubation, whereas treatment with 10 μM F-ara-A for 48 h did not cause any significant decrease in MCL-1 (Figure 6A). The exact mechanisms by which PEITC causes a decrease of MCL-1 remain unclear at the present time. One possible mechanism is that PEITC might induce degradation of MCL-1 protein due to a severe depletion of glutathione, leading to lower glutathionylation of MCL-1 protein and instability. Indeed, our previous study showed that MCL-1 protein was glutathionylated in CLL cells and such glutathionylation was reduced by PEITC treatment [13]. The fact that addition of NAC to replenish GSH could increase the stability of MCL-1 and prevent PEITC-induced loss of MCL-1 [13] further support the importance of glutathionylation in the regulation of MCL-1 stability. Interestingly, a recent study showed that inactivation of MCL-1 by a NOXA-dependent pathway sensitized renal cancer cells to chemotherapeutic agents [25]. The involvement of NOXA in affecting MCL-1 stability in CLL cells and the underlying mechanisms remains to be investigated.

In summary, our study showed that although CLL cells with loss of p53 were resistant to conventional anti-CLL drugs such as F-ara-A, Oxaliplatin and Bendamustine, these leukemia cells remain sensitive to PEITC, even when CLL cell was under the protection of bone marrow stromal cells. This high sensitivity of CLL cells to ROS-modulating agents is likely due to the intrinsic oxidative stress when p53 function is lost in CLL cells as a consequence of 17p-deletion. PEITC induced massive cell death in primary 17p- CLL cells and p53^−/−^ mouse leukemia cells through induction of severe GSH depletion and ROS accumulation, accompanied by a decrease of MCL-1 protein. Most importantly, *in vivo* treatment *TCL1*-Tg:*p53*^−/−^ CLL with PEITC significantly extended the survival time of the leukemic mice. It should be noted that PEITC at the concentration of 5 μM was highly effective against p53^−/−^ CLL cells, and such concentration range is likely achievable *in vivo* according to pharmacokinetic study [26, 27]. The promising therapeutic activity of PEITC against p53-defficient CLL cells *in vitro* and *in vivo* suggests that this compound has a potential for use in treatment of CLL patients with 17p-deletion, and warrants further evaluation in clinical setting.

## MATERIALS AND METHODS

### Reagents

PEITC, F-ara-A, N-acetylcysteine (NAC), Oxaliplatin, Propidium Iodide (PI) and Ethyl Acetate (Et Ac) were purchased from Sigma-Aldrich (St. Louis, MO). Pluronic^®^ F127 (F127) was purchased from Sigma (USA). Fludarabine used in the survival experiment was purchased from the Pharmacy of MD Anderson Cancer Center. PEITC was dissolved in Dimethyl Sulfoxide (DMSO) to make a 10 mM stock solution and PEITC working solution was freshly prepared by diluting the stock solution in culture medium. Ficoll-lite Lympho H was from Atlanta Biological (Lawrenceville, GA). CD19 microbeads were purchased from MACS Miltenyi Biotech Inc. (Auburn, CA). ACK lysis buffer and Annexin V-FITC were from BD Biosciences (San Jose, CA). CM-H2DCF-DA was purchased from Invitrogen (Carlsbad, CA). Terminal deoxynucleotidyl transferase deoxyuridine-triphosphatase nick-end labeling (TUNEL) staining kit was purchased from Roche Applied Science (Indianapolis, IN). A glutathione assay kit was purchased from Cayman Chemical (Ann Arbor, MI). Anti-MCL-1 was from Santa Cruz Biotechnology (Santa Cruz, CA). Anti-LC3 was purchased from EMD Millipore Corporation (Billerica, MA). Anti-β-actin was purchased from Cell Signaling Technology Inc. (Danvers, MA).

### Formulation of PEITC or *in vivo* administration

PEITC (20 mg) was dissolved in EtAc (1 ml) to form PEITC solution, which was introduced into 2 mL of pluronic F127 water solution at the concentration of 5% (w/w) under stirring by T10 homogenizer (IKA, Germany) in ice bath. About 30 min later, well-mixed oil in water (O/W) emulsion was formed. Then, EtAc was evaporated in rotator evaporator (BÜCHI, Switzerland) at 4°C for 4–6 hours and the PEITC nanoparticles were obtained. The PEITC nanoparticles slurry was lyophilized and the powder was stored at 4°C for future use. All the procedures were performed in the environment direct light exposure. *TCL1*-Tg:*p53*^−/−^ mice were treated with (100 mg/kg) formulated PEITC twice a week through tail vein injection (i.v.). Treatment began when the mice reached the age of 8 weeks.

### Isolation of CLL cells and cytotoxicity assays

Primary leukemia cells (white blood cells) were isolated from the peripheral blood samples of CLL patients diagnosed according to the NCI criteria [28]. Proper informed consents under a research protocol approved by the Institutional Review Board (IRB) of MD Anderson Cancer Center were obtained from all patients before the collection of blood samples. Primary CLL cells were isolated from blood samples by density gradient centrifugation as described previously [13], and incubated in RPMI 1640 medium supplemented with 10% FBS and Penicillin (100 U/ml) + Streptomycin (100 ug/ml) overnight before testing drug sensitivity by incubation with F-ara-A or Oxaliplatin for 48 h or PEITC for 24 h in presence or absence of human stromal NKTert cells. CD19+ B cells were purified from CLL blood samples by using CD19 microbeads, and incubated in RPMI 1640 medium supplemented with 10% FBS and Penicillin (100 U/ml) + Streptomycin (100 ug/ml). On the same day, these B leukemic cells were co-cultured with stromal NKTert cells and treated with F-ara-A or Oxaliplatin for 48 h or PEITC for 24 h. Mouse splenocytes were isolated from *TCL1*-Tg:*p53*^−/−^ mice, and red blood cells were removed from the splenocytes by ACK lysis buffer. After lysis of red blood cells, the splenocytes were washed with RPMI1640 culture medium and PBS. On the same day of isolation, the mouse splenocytes were co-cultured with murine stromal cells (Kusa-H1) and treated with F-ara-A or Oxaliplatin for 48 h or PEITC for 24 h. Cell viability and cellular sensitivity to drug treatment *in vitro* were determined by flow cytometry after double staining of 1 × 10^6^ cells with annexinV-FITC and PI as previously described [29].

### TUNEL assay

*TCL1*-Tg:*p53*^−/−^ mice were generated and maintained as described [10]. Mouse spleen sections were fixed in neutral buffered containing 10% formalin solution for preparation of tissue slides. TUNEL assays were performed with an *In Situ* Cell Death Detection kit (Roche) according to manufacturer's instruction, and visualized using a fluorescent microscope.

### Immunoblotting

MCL-1 protein levels were determined by Western blotting as described [10].

### Detection of ROS

Cellular ROS contents were measured by incubating CLL cells (5 × 10^5^ cells) with 1 μM CM-H2DCF-DA for 60 minutes and analyzed by flow cytometry as described [21].

### Analysis of cellular glutathione

A glutathione assay kit (Cayman Chemical, Ann Arbor, MI) was used to measure cellular glutathione. After preparing cell extracts by sonication and deproteination, GSH was determined as described [21].

### Statistical analysis

Student's *t* test was used for testing the statistical difference between two groups of samples. Mouse survival curves (Kaplan-Meier plots) were generated by Graphpad Prism software (GraphPad, San Diego, CA) as described [10], and the statistical significance of the mouse survival curves was analyzed by the log-rank (Mantel-Cox) test. A *p value* of less than 0.05 was considered statistically significant.

## SUPPLEMENTARY MATERIALS FIGURES


