# Supplementary material for: Targeting p53-deficient chronic lymphocytic leukemia cells in vitro and in vivo by ROS-mediated mechanism
Source: Oncotarget. 2016 Sep 19;7(44):71378–89. doi: 10.18632/oncotarget.12110 (PMC5342085; doi:10.18632/oncotarget.12110)
Supplement: Supplementary file 1 [file oncotarget-07-71378-s001.pdf]

Targeting p53-deficient chronic lymphocytic leukemia cells *in vitro* and *in vivo* by ROS-mediated mechanism

Supplementary Materials

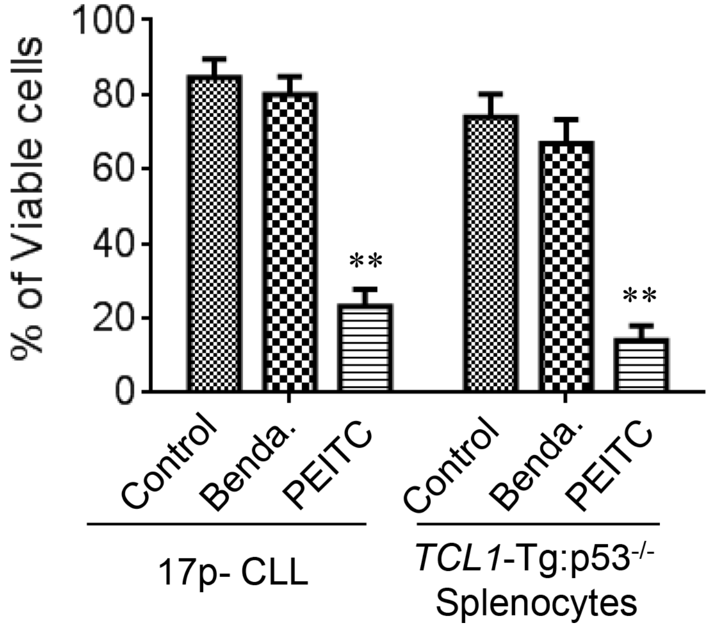

Supplementary Figure S1: Effect of PEITC and Bendamustine on 17p- CLL cells. Primary CLL cells with 17p deletion were co-cultured with NKTert stromal cells, and leukemic splenocytes isolated from *TCL1*-Tg:p53$^{-/-}$ mice were co-cultured with mouse stromal Kusa.H1 cells. The samples were treated with 10 µM Bendamustine or 5 µM PEITC for 24 h as indicated. Cell viability was measured by flow cytometry analysis after double staining with Annexin-V/PI. $^{**}p < 0.01$; $n = 6$.

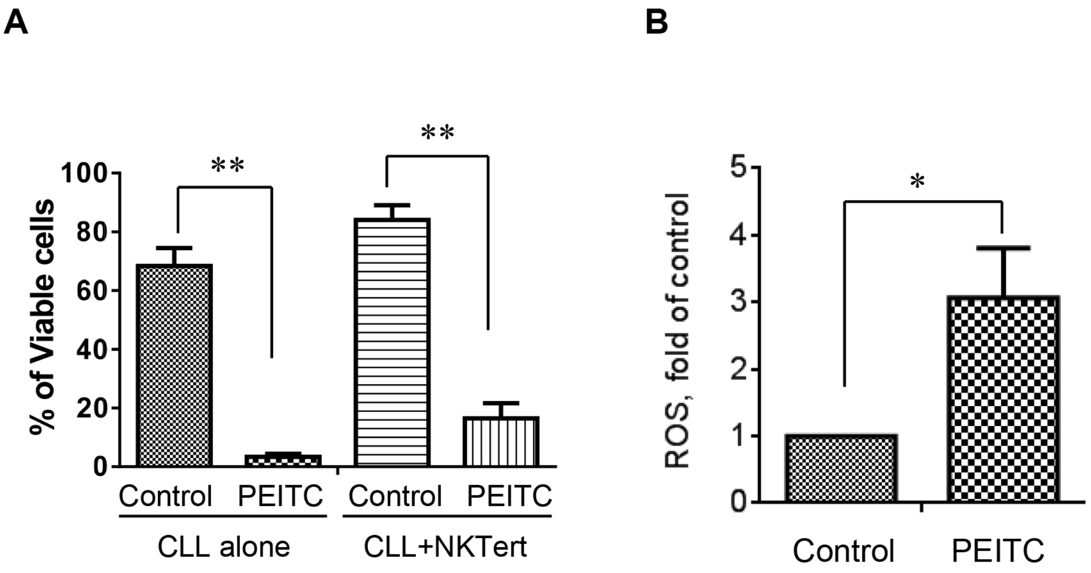

Supplementary Figure S2: Effect of PEIT on cell viability and cellular ROS in CLL cells without 17p deletion. Bar graphs showing quantitative analysis of cell death (**A**) and ROS levels (**B**). CLL cells were treated with 5 µM PEITC for 24 h. Cell viability was measured by flow cytometry after double staining with Annexin-V/PI ($n = 6$). Cellular ROS was analyzed by flow cytometry after staining with DCF-DA 1.5 hours after PEITC treatment ($n = 6$). *$p < 0.05$, **$p < 0.01$.

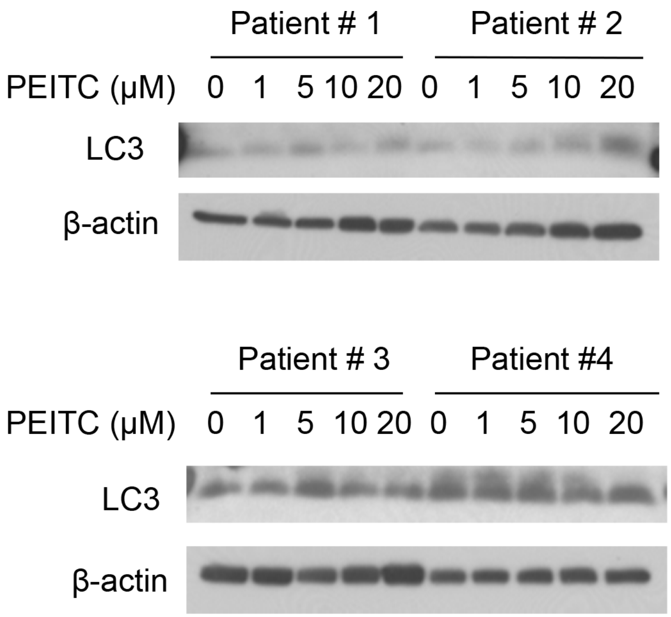

Supplementary Figure S3: Effect of PEITC on the expression of autophagy marker LC3 in CLL cells. LC3 levels were measured by Western blotting in CLL cells before and after treatment with various concentrations of PEITC for 24 h as indicated. Note: Patient #2 had 17p-deletion; patients #1, #3, and #4 were without 17p-deletion.
